# Dental and Medical Infection

**Published:** 1896-12

**Authors:** 


THE
AMERICAN JOURNAL
—OF—
DENTAL SCIENCE.

Vol. xxx. Baltimore, December, 1896. No. 8.

ARTICLE I.

### DENTAL AND MEDICAL INFECTION.

In the July (1895) number of the *Montreal Medical Journal* an article appeared with the rather sensational heading, "Infection in the Dentist's Chair." descriptive of the case of a housemaid, who was admitted to the General Hospital a month after she had three teeth extracted. She complained of "sore throat and sore gums, and tender, painful teeth," not a very rare experience, under the circumstances. A week after she was admitted, she died. The case was reported—as the result of the autopsy—as one of septic infection, and without actually knowing anything whatever of the facts as to the condition of the instrument used by the dentist, and without investigating the circumstances preceding the girl's entrance to the hospital, the startling accusation was made that the infection was caused "in the dentist's chair." A reply was sent to the editor, but it never reached him.

Reasoning from analogy, the author of the reply endeavored to show that the possibility of infection in the dentist's chair, or from the dentist, was very much less than from the general physician, especially if he practiced surgery, and from general hospitals; and an article by Professor Fournier, of Paris, in a recent medical journal, so well expresses and exposes this position that we take the liberty of making extensive quotations. It is an undeniable fact that physicians do infect patients in various ways—through the hands, through instruments, through transportation of organic substances from syphilitic organisms, and through the clothes. Professor Fournier's article is confined to syphilitic infection. He shows that by digital examinations—and quotes facts—that syphilis is conveyed by direct transportation, as it were, and refers to an epidemic of syphilis in the eighteenth century which originated through a syphilitic midwife, who continued practice despite the lesions upon her hand. Referring to instruments, Professor Fournier specially blames the bistoury, the lancet, the accessories used in applying simple or scarifying cups (glasses, razor and scariffier), the probe, the speculum, the Eustachian catheter, the tongue depressor, the laryngoscope, and the various articles used in surgical dressings—lints, sponges, linens, etc.

"Side by side," says the writer, "with the speculum may be placed the tongue depressor. The mouth is examined on all possible occasions in dental diseases, in throat maladies, etc., and thus may become the focus *par excellence* of syphilitic contagion; the least inattention may prove disastrous. After examining the oral cavity of a syphlitic, full perhaps of mucous plaques, the tongue depressor is laid aside without cleaning, and is forgotten; another patient comes in whose mouth is examined by means of the same implement, and infection is the result." Professor Fournier exposes, too the dangers from the use of nitrate of silver pencils, which are now interdicted in French hospitals. Skin-grafting, vaccination, etc., come in for their share of condemnation in the same relation.

Quite as important as any causes of infection, are those which occur from the physician to the patient, and *vice versa*. The physician may be infected in the face and in the hands by direct contact with the contaminating pus, or by contact with the globules of sputum projected from the mouth or throat of the patient. Physicians are exposed to a "veritable rain of salivary globules" while cauterizing the throats of patients, as small-pox and syphilis have both been contracted in this way. "Manual chancre is the medical chancre *par excellence.* This may arise during operations on the penis, vaginal examinations, obstetrical manoeuvres, operations on syphilitic subjects, also wounds received during autopsies.

It is a sad and startling fact, that professional syphilis is not uncommon, contracted in practice. It appears to be more dangerous to life than that ordinarily contracted, because, as Professor Fournier argues, the physician is morally depressed, is overdriven, and is inadequately treated. "A man of the world may contract syphilis, become pre-occupied and wretched, but we can console and reassure him by all manner of specious arguments. The physician, on the contrary, knows too well what the malady means, and the danger that will menace him in the future."

The possibility of infection of various kinds from house to house conveyed by the family physicians; the difficulty of excluding it invariably from hospital practice; the slovenliness of some practitioners, who proceed from surgical to obstetrical cases, and whose ether and instrument bags will frequently not bear inspection, which the chair, the instruments, the linen, or the person of the average dentist will stand, should make medical critics of "dental infection" think twice and investigate fully before, from glass houses, they throw stones.—*Editorial in Dominion Dental Journal.*

